# NT-PGC-1α deficiency attenuates high-fat diet-induced obesity by modulating food intake, fecal fat excretion and intestinal fat absorption

**DOI:** 10.1038/s41598-020-79823-9

**Published:** 2021-01-14

**Authors:** Jihyun Kim, Jiyoung Moon, Chul-Hong Park, Jisu Lee, Helia Cheng, Z. Elizabeth Floyd, Ji Suk Chang

**Affiliations:** 1 Laboratory of Gene Regulation and Metabolism, Pennington Biomedical Research Center, 6400 Perkins Road, Baton Rouge, LA 70808 USA; 2 Laboratory of Ubiquitin Biology, Pennington Biomedical Research Center, Baton Rouge, LA USA

**Keywords:** Fat metabolism, Obesity

## Abstract

Transcriptional coactivator PGC-1α and its splice variant NT-PGC-1α regulate metabolic adaptation by modulating many gene programs. Selective ablation of PGC-1α attenuates diet-induced obesity through enhancing fatty acid oxidation and thermogenesis by upregulation of NT-PGC-1α in brown adipose tissue (BAT). Recently, we have shown that selective ablation of NT-PGC-1α reduces fatty acid oxidation in BAT. Thus, the objective of this study was to test our hypothesis that NT-PGC-1α^−/−^ mice would be more prone to diet-induced obesity. Male and female NT-PGC-1α^+/+^ (WT) and NT-PGC-1α^−/−^ mice were fed a regular chow or 60% high-fat (HF) diet for 16 weeks. Contrary to our expectations, both male and female NT-PGC-1α^−/−^ mice fed HFD were protected from diet-induced obesity, with more pronounced effects in females. This lean phenotype was primarily driven by reduced dietary fat intake. Intriguingly, HFD-fed female, but not male, NT-PGC-1α^−/−^ mice further exhibited decreased feed efficiency, which was closely associated with increased fecal fat excretion and decreased uptake of fatty acids by the intestinal enterocytes and adipocytes with a concomitant decrease in fatty acid transporter gene expression. Collectively, our results highlight the role for NT-PGC-1α in regulating whole body lipid homeostasis under HFD conditions.

## Introduction

The *PPARGC1A* gene produces two major isoforms of transcriptional coactivators with distinct protein structure, a full-length PGC-1α (797 aa) and a shorter isoform NT-PGC-1α (270 aa)^1^. Although NT-PGC-1α lacks the C-terminal domain (271–797 aa) of PGC-1α, it can co-activate a number of nuclear receptors through its transcription activation and nuclear receptor interaction domains, leading to transcriptional activation of various genes involved in mitochondrial oxidative metabolism and thermogenesis, lipid metabolism, glycolysis, gluconeogenesis, and angiogenesis^1–7^. PGC-1α and NT-PGC-1α play shared and distinct roles in regulating diverse tissue-specific transcription programs and work in concert to mediate cellular adaptation to various physiological stimuli, such as environmental temperature, exercise, and fasting^1–4,6–9^.

PGC-1α^−/−^ mice lacking both PGC-1α and NT-PGC-1α are unable to maintain body temperature during cold exposure due to impaired BAT thermogenesis^10^. Despite having the reduced thermogenic capacity, PGC-1α^−/−^ mice are resistant to diet-induced obesity because of profound hyperactivity associated with abnormal CNS function. On the contrary, mice selectively lacking full-length PGC-1α (also designated as FL-PGC-1α) are cold-tolerant^4,5^. This is largely due to compensatory elevation of NT-PGC-1α in FL-PGC-1α^−/−^ BAT, resulting in enhanced fatty acid oxidation and thermogenesis^4,11^, which in turn contributes to the protection of FL-PGC-1α^−/−^ mice from diet-induced obesity^11^. Recently, we generated NT-PGC-1α^−/−^ mice selectively lacking NT-PGC-1α but expressing FL-PGC-1α^12^ and showed that NT-PGC-1α ablation decreases fatty acid oxidation in BAT, leading to a greater reliance on glucose oxidation for thermogenesis.

Given this defect in fatty acid oxidation in BAT, we investigated if NT-PGC-1α^−/−^ mice are prone to HFD-induced obesity. Male and female NT-PGC-1α^+/+^ (WT) and NT-PGC-1α^−/−^ mice were fed a regular chow diet or a 60% fat diet for 16 weeks. Contrary to our expectations, we found that both male and female NT-PGC-1α^−/−^ mice fed HFD display a significant reduction in weight gain compared to WT mice primarily due to decreased dietary fat intake. Intriguingly, female NT-PGC-1α^−/−^ mice exhibited the greater resistance to diet-induced obesity; this was associated with increased fecal fat excretion, decreased intestinal fat absorption, and decreased fatty acid uptake by the adipose tissue. Taken together, we have identified NT-PGC-1α as a pivotal regulator of whole body lipid homeostasis under HFD conditions.

## Results

### NT-PGC-1α ablation attenuates high-fat diet-induced obesity

**Figure 1:**
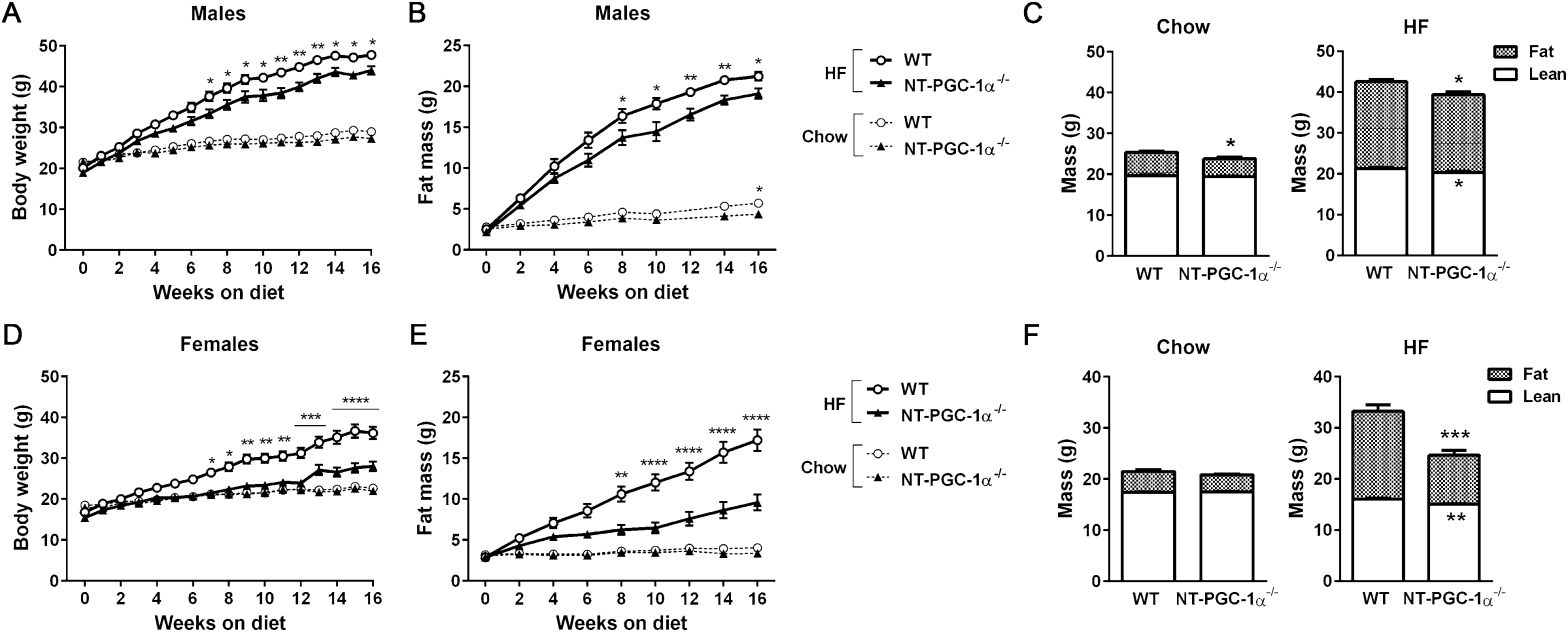
Analysis of the effects of NT-PGC-1α ablation on diet-induced obesity. (**A**,**B**) Measurements of body weight and fat mass of male WT and NT-PGC-1α^−/−^ mice during chow (n = 11 per group) or HFD (n = 12 per group) feeding. Two-way ANOVA was used to determine the differences between the genotypes. BW: F (1, 24) = 9.726, *P* = 0.0048; FM: F (1, 22) = 5.939, *P* = 0.0234 in HFD-fed group. BW: F (1, 22) = 3.485, *P* = 0.0753; FM: F (1, 20) = 5.843, *P* = 0.0253 in chow-fed group. (**C**) Body composition of male WT and NT-PGC-1α^−/−^ mice after 16 weeks of either chow- or HFD-feeding. Data are presented as the mean ± SEM. *P < 0.05 determined by Student’s *t* test. (**D**,**E**) Body weight and fat mass of female WT and NT-PGC-1α^−/−^ mice during chow (n = 11 per group) or HFD (n = 12 per group) feeding. Two-way ANOVA was used to determine the differences between the genotypes. BW: F (1, 24) = 20.50, *P* = 0.0001; FM: F (1, 22) = 21.34, *P* = 0.0001 in HFD-fed group. BW: F (1, 22) = 0.7853, *P* = 0.3851; FM: F (1, 20) = 2.426, *P* = 0.1350 in chow-fed group. (**F**) Body composition of female WT and NT-PGC-1α^−/−^ mice after 16 weeks of either chow- or HFD-feeding. Data are presented as the mean ± SEM. **P < 0.01, ***P < 0.001 determined by Student’s *t* test.

To test our prediction that NT-PGC-1α^−/−^ mice would be prone to HFD-induced obesity, male and female NT-PGC-1α^+/+^ (WT) and NT-PGC-1α^−/−^ mice were fed a chow or high-fat diet (HFD) for 16 weeks starting at 5 weeks of age. On a chow diet, both male and female WT and NT-PGC-1α^−/−^ mice showed no difference in weight gain during the course of study (Fig. 1A,D, dotted lines). However, contrary to our prediction, when placed on a HFD, both male and female NT-PGC-1α^−/−^ mice gained weight at a significantly slower rate than WT mice (Fig. 1A,D, solid lines). Moreover, the difference in weight gain between WT and NT-PGC-1α^−/−^ mice was more pronounced in females. After 16 weeks of HFD feeding, NT-PGC-1α^−/−^ females exhibited a 35% reduction in weight gain compared to WT control mice, whereas NT-PGC-1α^−/−^ males exhibited a 10% reduction (Fig. 1A,D). Body composition analyses further showed that male and female NT-PGC-1α^−/−^ mice gained substantially less fat than WT mice on the HFD (Fig. 1B,C,E,F), demonstrating that decreased body weight in NT-PGC-1α^−/−^ mice was primarily due to decreased accumulation of fat mass over the course of HFD feeding.

### Improved glucose tolerance, but not insulin sensitivity, in NT-PGC-1α^−/−^ mice fed HFD

**Figure 2:**
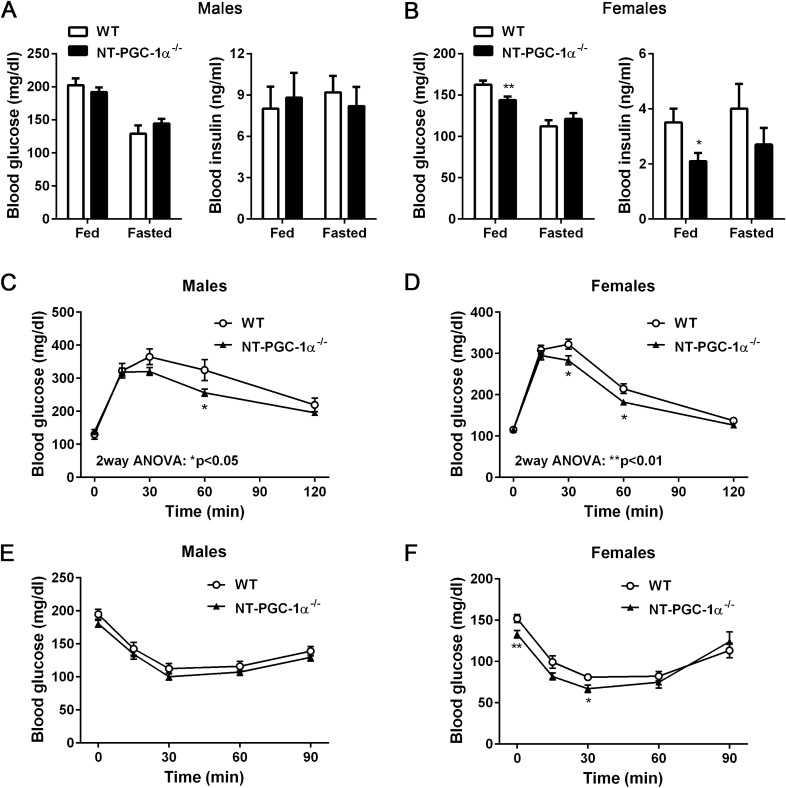
Whole body glucose homeostasis in NT-PGC-1α^−/−^ mice fed HFD. (**A**,**B**) Blood glucose and insulin levels in HFD-fed male and female WT and NT-PGC-1α^−/−^ mice in the fed and fasted states (n = 9 per group). Data are presented as the mean ± SEM. *P < 0.05, **P < 0.001 determined by Student’s *t* test. (**C**,**D**) Glucose tolerance test in male and female WT and NT-PGC-1α^−/−^ mice fed HFD for 9 weeks (n = 11–12 per group) in the overnight-fasted state. Two-way ANOVA was used to determine the differences between the genotypes. Males: F (1, 105) = 6.009, *P* = 0.0159; females: F (1, 110) = 11.15, *P* = 0.0011. (**E**,**F**) Insulin tolerance test in male and female WT and NT-PGC-1α^−/−^ mice fed HFD for 10 weeks in the 5 h-fasted state (n = 11–12 per group).

Given that decreased adiposity is often associated with improved glucose homeostasis, we examined whether decreased fat mass in HFD-fed NT-PGC-1α^−/−^ mice are associated with improved glucose handling and insulin sensitivity. Male NT-PGC-1α^−/−^ mice showed no difference in blood glucose and insulin levels compared to WT mice in both the fed and fasted states (Fig. 2A). In contrast, female NT-PGC-1α^−/−^ mice exhibited lower levels of blood glucose and insulin compared to WT mice in the fed state, but fasted blood glucose and insulin levels did not differ between the genotypes (Fig. 2B). During glucose tolerance tests, both male and female NT-PGC-1α^−/−^ mice exhibited improved glucose tolerance compared to WT mice (Fig. 2C,D). However, WT and NT-PGC-1α^−/−^ mice showed similar glucose disposal rates after insulin administration in both sexes (Fig. 2E,F).

### HFD-fed NT-PGC-1α^−/−^ female mice exhibit decreased feed efficiency associated with increased fecal triglyceride excretion

**Figure 3:**
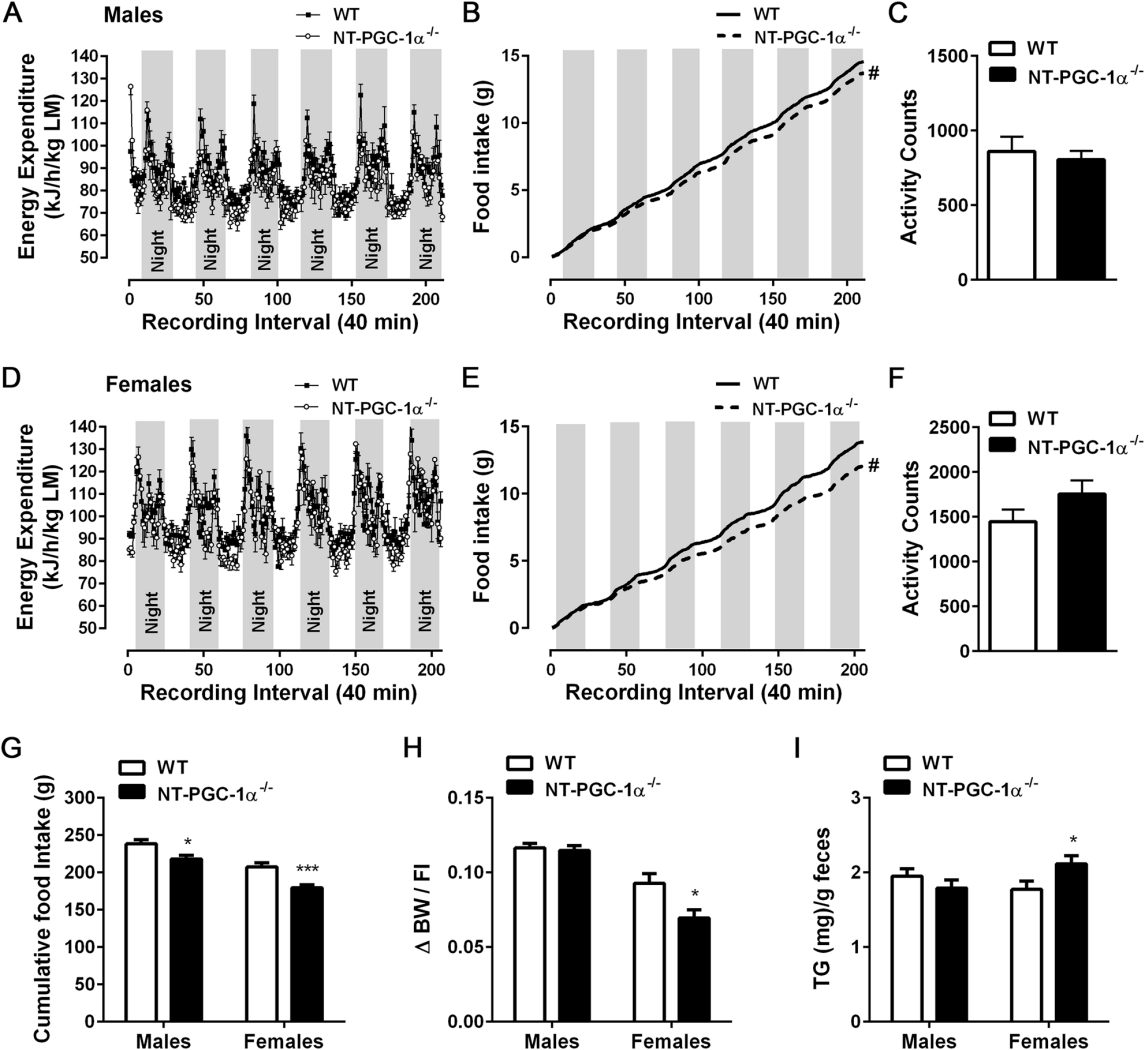
Caloric intake and energy metabolism in NT-PGC-1α^−/−^ mice fed HFD. (**A**–**C**) Energy expenditure, food intake, and average locomotor activity of male WT and NT-PGC-1α^−/−^ mice fed HFD (n = 11–12 per group). (**D**–**F**) Energy expenditure, food intake, and average locomotor activity of female WT and NT-PGC-1α^−/−^ mice fed HFD (n = 11–12 per group). Two-way ANOVA was used to determine the differences in food intake between the genotypes. Males: F (1, 4642) = 23.33, ^#^*P* < 0.0001; females: F (1, 4326) = 834.7, ^#^*P* < 0.0001. (**G**) Cumulative food intake of male and female WT and NT-PGC-1α^−/−^ mice during HFD feeding (n = 12 per group). Food intake was monitored once a week for 16 weeks and was expressed as g/mouse. (**H**) Cumulative weight (g) gained over 16 weeks divided by the cumulative food intake (g/mouse) over the same period on HFD (n = 12 per group). (**I**) Triglyceride content in feces from male and female WT and NT-PGC-1α^−/−^ mice fed HFD (n = 10 per group). All data are presented as the mean ± SEM. *P < 0.05, ***P < 0.001 determined by Student’s *t* test.

To explore possible factors contributing to the protection from diet-induced obesity in NT-PGC-1α^−/−^ mice, we analyzed parameters of energy balance. Indirect calorimetry analyses revealed that WT and NT-PGC-1α^−/−^ mice of both sexes exhibited comparable levels of energy expenditure and locomotor activity (Fig. 3A,C,D,F). Instead, both male and female NT-PGC-1α^−/−^ mice ate less than WT mice on the HFD (Fig. 3B,E), and cumulative food intake per mouse over 16 weeks was considerably lower in NT-PGC-1α^−/−^ mice compared with WT mice (Fig. 3G). This reduction in food intake in NT-PGC-1α^−/−^ mice was only observed under HFD conditions. Chow-fed WT and NT-PGC-1α^−/−^ mice of both sexes exhibited no differences in energy expenditure, locomotor activity, and food intake (see Supplementary Fig. S1 online).

We calculated feed efficiency (body weight gain/food eaten) to determine the weight gained per gram of food consumed over 16 weeks. Feed efficiency was comparable between male WT and NT-PGC-1α^−/−^ mice (Fig. 3H), indicating that decreased dietary fat intake fully accounts for lower weight gain in male NT-PGC-1α^−/−^ mice. However, surprisingly, feed efficiency was 25% less in female NT-PGC-1α^−/−^ mice compared to WT controls (Fig. 3H). We thus explored the potential effect of NT-PGC-1α ablation on fecal energy excretion by measuring fecal lipid content. While fecal triglyceride (TG) content did not differ between male WT and NT-PGC-1α^−/−^ mice, fecal TG content was ~ 20% greater in female NT-PGC-1α^−/−^ mice than in WT controls (Fig. 3I). Thus, these findings demonstrate that increased fecal TG excretion contributes to the greater resistance of female NT-PGC-1α^−/−^ mice to HFD-induced obesity.

### NT-PGC-1α^−/−^ adipocytes are resistant to HFD-induced hypertrophy and inflammation

**Figure 4:**
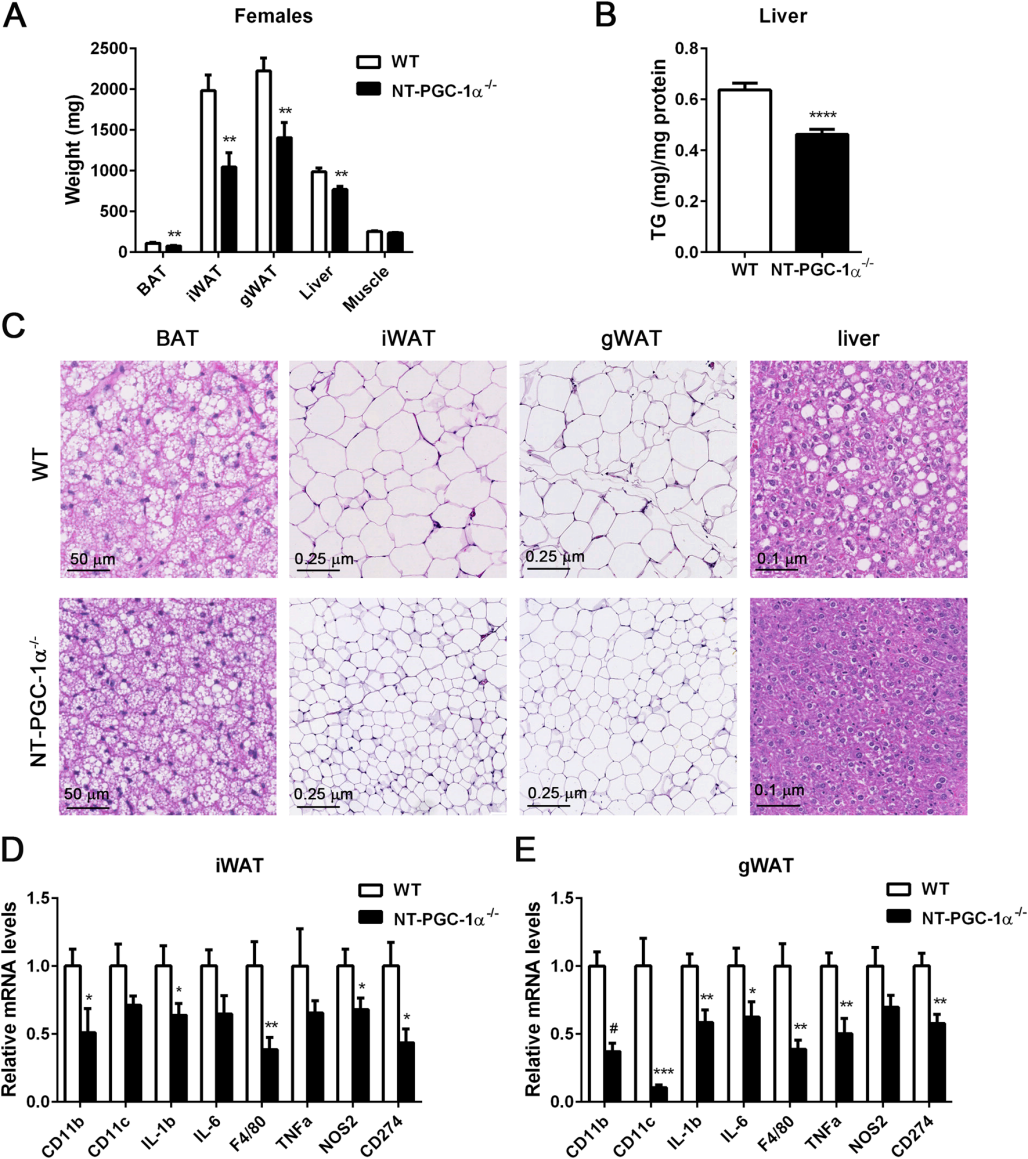
NT-PGC-1α ablation reduces HFD-induced adipocyte hypertrophy and inflammation. (**A**) Weights of tissues collected from female WT and NT-PGC-1α^−/−^ mice fed HFD for 16 weeks (n = 12 per group). (**B**) Triglyceride content of livers from female WT and NT-PGC-1α^−/−^ mice fed HFD (n = 10 per group). (**C**) Representative images of H&E-stained sections of adipose tissues and livers from female WT and NT-PGC-1α^−/−^ mice fed HFD. Images were viewed through the NDP.view 2 software (https://www.hamamatsu.com/us/en/product/type/U12388-01/index.html). Magnification ×16.1 (BAT), ×5 (iWAT, gWAT), and ×10 (liver). (**D**, **E**) Quantitative real time PCR analysis of genes involved in inflammation in inguinal and gonadal adipose tissue from WT and FL-PGC-1α^−/−^ female mice fed HFD (n = 10–12 per group). All data are presented as the mean ± SEM. *P < 0.05, **P < 0.01, ***P < 0.001, ****P < 0.0001 determined by Student’s *t* test.

Decreased dietary fat intake and increased fecal fat excretion in NT-PGC-1α^−/−^ female mice were closely associated with a large decrease in brown, inguinal, and gonadal fat pads (Fig. 4A). Histological analyses further revealed that adipocytes of brown, inguinal, and gonadal fat pads from NT-PGC-1α^−/−^ female mice were significantly smaller than adipocytes from WT mice (Fig. 4C). In line with the smaller adipocyte size, NT-PGC-1α^−/−^ inguinal and gonadal white adipose tissue (WAT) exhibited markedly decreased expression of M1 macrophage marker genes (CD11b, CD11c, F4/80, CD274) and pro-inflammatory genes (TNFα, IL6, IL1β, NOS2) compared to WT controls (Fig. 4D,E), demonstrating decreased macrophage infiltration into white adipose tissue, as previously shown^13–15^. Moreover, NT-PGC-1α^−/−^ female mice were protected from HFD-induced hepatic steatosis as evidenced by a decrease in weight, lipid droplet accumulation, and TG content in the liver (Fig. 4A–C).

### Fatty acid uptake is decreased in NT-PGC-1α^−/−^ adipose tissue

**Figure 5:**
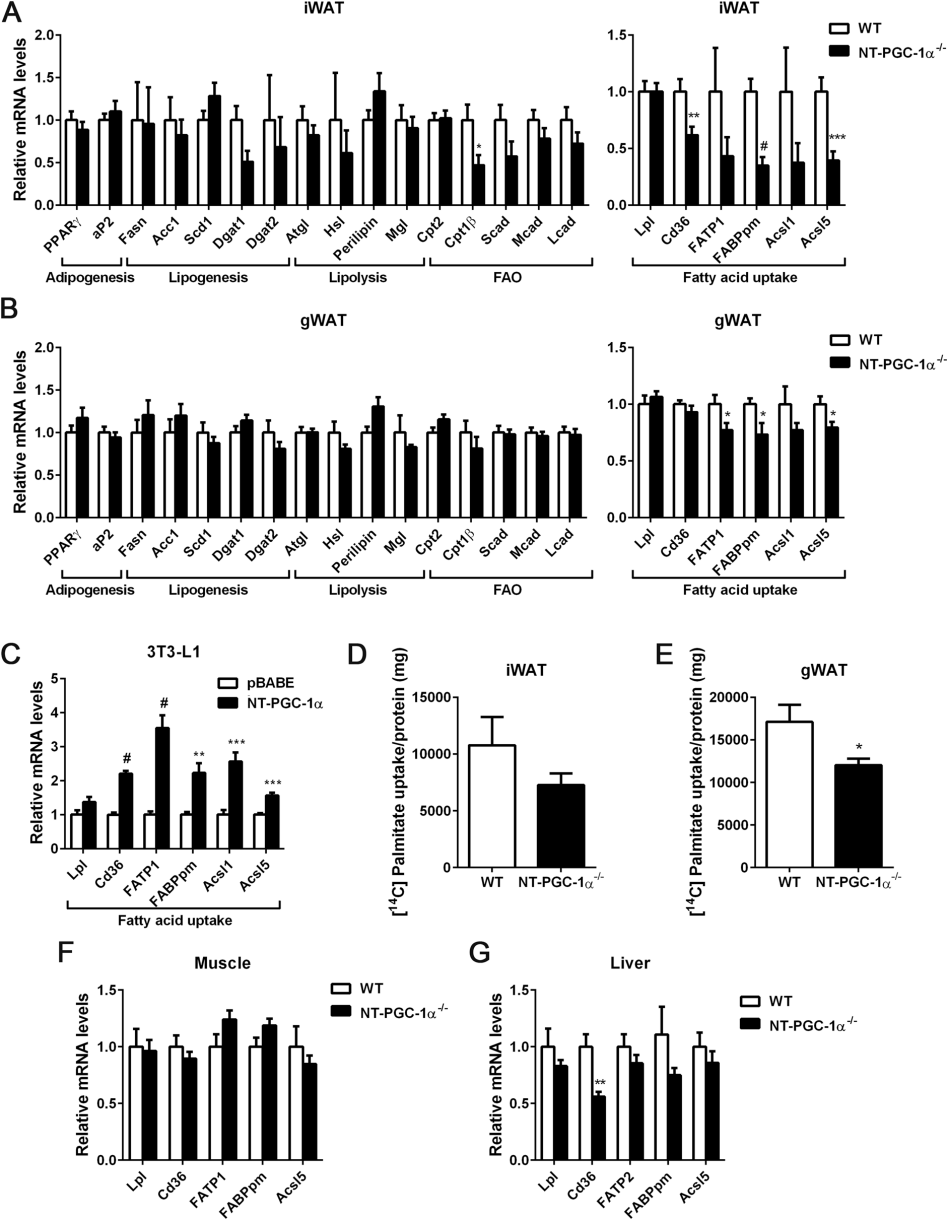
Decreased fatty acid uptake in adipose tissue from HFD-fed female NT-PGC-1α^−/−^ mice. (**A**, **B**) Quantitative real time PCR analysis in inguinal and gonadal adipose tissue from female WT and NT-PGC-1α^−/−^ mice fed HFD for 16 weeks (n = 12 per group). (**C**) Upregulation of fatty acid uptake genes by NT-PGC-1 in 3T3-L1 adipocytes (n = 6 per group). (**D**, **E**) Uptake of [^14^C]-palmitate in inguinal and gonadal adipose tissue from female WT and NT-PGC-1α^−/−^ mice fed HFD for 8 weeks (n = 6 per group). (**F**, **G**) Expression of fatty acid uptake genes in muscle and liver from female WT and NT-PGC-1α^−/−^ mice fed HFD for 16 weeks (n = 12 per group). All data are presented as the mean ± SEM. *P < 0.05, **P < 0.01, ***P < 0.001, ^#^*P* < 0.0001 determined by Student’s *t* test.

We next assessed whether altered adipose lipid metabolism (i.e. decreased lipogenesis, increased lipolysis, or increased fatty acid oxidation) contributes to the reduced adiposity in HFD-fed NT-PGC-1α^−/−^ female mice. Gene expression analyses of inguinal and gonadal WAT revealed that expression of genes involved in lipogenesis, lipolysis, and fatty acid oxidation is comparable between WT and NT-PGC-1α^−/−^ mice (Fig. 5A,B). In contrast, key genes encoding the fatty acid transporters (CD36, FATP1, FABPpm) and acyl-CoA synthase 5 (ACSL5), an enzyme catalyzing re-esterification of dietary fatty acids into triglyceride, were significantly downregulated in NT-PGC-1α^−/−^ inguinal and gonadal WAT (Fig. 5A,B). To determine if NT-PGC-1α transcriptionally regulates these genes critical for fatty acid uptake in white adipocytes, we expressed NT-PGC-1α in differentiated 3T3-L1 adipocytes. Indeed, NT-PGC-1α elevated the expression of fatty acid transporters (CD36, FATP1, FABPpm) and long-chain fatty-acid-coenzyme A ligases (ACSL1, ACSL5) in adipocytes (Fig. 5C). Consistent with decreased expression of fatty acid transporter genes in NT-PGC-1α^−/−^ adipose tissue, inguinal WAT isolated from HFD-fed NT-PGC-1α^−/−^ female mice showed a trend toward lower [^14^C]-palmitate uptake (Fig. 5D) and gonadal NT-PGC-1α^−/−^ WAT showed a ~ 30% decrease in [^14^C]-palmitate uptake compared with WT controls (Fig. 5E).

There was no alteration in fatty acid transporter gene expression in NT-PGC-1α^−/−^ muscle (Fig. 5F), which is correlated with no change in muscle weight (Fig. 4A). However, NT-PGC-1α^−/−^ liver showed decreased CD36 expression (Fig. 5G), which is closely associated with a decrease in liver weight and hepatic TG content (Fig. 4B,C). This observation is consistent with previous findings that HFD-dependent elevation of CD36 expression in the liver increases fatty acid uptake and contributes to HFD-induced hepatic TG storage^16,17^. Taken together, these results indicate that NT-PGC-1α regulates HFD-induced uptake of fatty acids in adipose tissue and possibly in liver.

### Intestinal fatty acid absorption is reduced in HFD-fed NT-PGC-1α^−/−^ female mice

**Figure 6:**
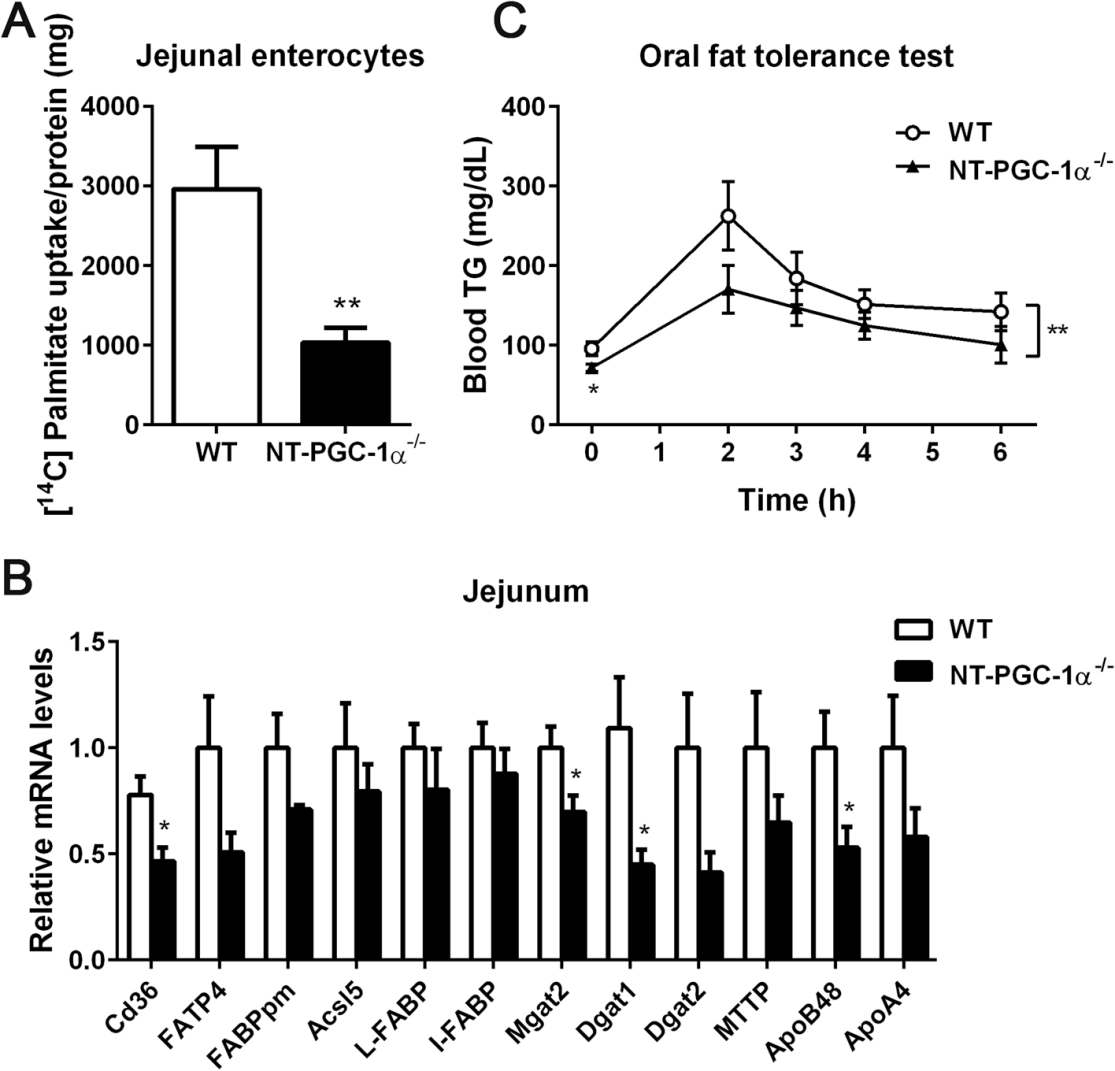
Reduced intestinal lipid absorption in NT-PGC-1α^−/−^ female mice fed HFD. (**A**) Uptake of [^14^C]-palmitic acids in jejunal enterocytes isolated from female WT and NT-PGC-1α^−/−^ mice fed HFD (n = 5 per group). Data are presented as the mean ± SEM. **P < 0.01 determined by Student’s *t* test. (**B**) Quantitative real time PCR analysis of genes involved in intestinal fat absorption and secretion in the jejunum of female WT and NT-PGC-1α^−/−^ mice fed HFD (n = 6–7 per group). Data are presented as the mean ± SEM. *P < 0.05 determined by Student’s *t* test. (**C**) Oral fat tolerance test. Female WT and NT-PGC-1α^−/−^ mice fed HFD for 8 weeks were fasted overnight and administered by gavage of olive oil. Blood samples were collected at indicated times and analyzed for serum TG concentrations (n = 7 per group). Two-way ANOVA was used to determine the differences in serum TG levels between the genotypes. F (1, 60) = 7.941, **P = 0.0065.

Chronic high-fat diet has been shown to increase intestinal, particularly jejunal, lipid absorption in mice^18^. Intestinal lipid absorption involves hydrolysis of dietary triglycerides to free fatty acids and 2-monoacylglycerols in the intestinal lumen^19,20^. These hydrolysis products are then taken up by enterocytes, followed by re-synthesis to triglycerides and packaging into chylomicrons by microsomal triglyceride transfer protein (MTP) for secretion into the circulation^19,20^. It has been shown that PGC-1α, which represents both PGC-1α and NT-PGC-1α, is expressed in the apical compartment of the intestinal epithelium and regulates mitochondrial biogenesis and respiration in enterocytes^21^. Given that HFD-fed NT-PGC-1α^−/−^ female mice exhibited decreased feed efficiency (Fig. 3H), we examined if intestinal fatty acid absorption is decreased in HFD-fed NT-PGC-1α^−/−^ female mice. To do this, we performed fatty acid uptake assays using enterocytes freshly isolated from the jejunum of HFD-fed WT and NT-PGC-1α^−/−^ female mice. Strikingly, NT-PGC-1α^−/−^ jejunal enterocytes exhibited a ~ 65% decrease in [^14^C]-palmitate uptake compared with WT controls (Fig. 6A). Consistent with this decrease, genes encoding fatty acid transporters (CD36, FATP4, FABPpm), which are highly expressed on the luminal surface of the small intestine^22,23^ and facilitate the protein-mediated transport of fatty acids across the apical membrane of the enterocytes^24^, were downregulated in the jejunum of HFD-fed NT-PGC-1α^−/−^ female mice (Fig. 6B). Moreover, expression of key genes that regulate intestinal TG synthesis (MGAT2, DGAT1, DGAT2), packaging and secretion of chylomicrons (MTP, ApoB48, ApoA4) was largely decreased in the jejunum of HFD-fed NT-PGC-1α^−/−^ female mice compared to WT controls (Fig. 6B). This is in line with the previous findings that CD36 deficiency subsequently impairs induction of genes of intestinal chylomicron formation and secretion^25,26^. Together, these results indicate that decreased intestinal fatty acid absorption contributes to the greater resistance of female NT-PGC-1α^−/−^ mice to HFD-induced obesity.

We next carried out an oral fat tolerance test (OFTT) to assess the effect of a high lipid load on circulating TG levels in HFD-fed WT and NT-PGC-1α^−/−^ female mice. WT and NT-PGC-1α^−/−^ female mice fed HFD for 8 weeks were fasted overnight and gavaged with an olive oil bolus, and serum TG levels were determined before (T0) and after (T2, T3, T4, and T6) the lipid challenge. As expected, NT-PGC-1α^−/−^ female mice exhibited lower serum TG levels than WT control mice at T0 (Fig. 6C). In addition, an acute rise in serum TG levels from T0 to T2 was lower in NT-PGC-1α^−/−^ female mice and the downward slope of TG levels from T2 to T6, which reflects the speed of TG clearance from the circulation, was lower in NT-PGC-1α^−/−^ female mice compared to WT control mice (Fig. 6C). It is unlikely that decreased serum TG levels were caused by efficient clearance of circulating TG by peripheral tissues in NT-PGC-1α^−/−^ mice because HFD-fed NT-PGC-1α^−/−^ female mice displayed reduced fatty acid uptake in adipose tissue and decreased TG accumulation in the liver. Collectively, this result shows that NT-PGC-1α is an important player in regulating whole body lipid homeostasis under HFD conditions.

## Discussion

We previously showed that NT-PGC-1α ablation decreases fatty acid oxidation in BAT^12^. Thus, we hypothesized that NT-PGC-1α^−/−^ mice would be more prone to diet-induced obesity under high fat dietary conditions. Contrary to our expectations, the results of our studies revealed that both male and female NT-PGC-1α^−/−^ mice were protected from HFD-induced obesity with more pronounced effects in females. This lean phenotype was not due to alteration in energy expenditure or locomotor activity and was instead driven by reduced food intake. Surprisingly, our study further revealed that NT-PGC-1α^−/−^ mice exhibit a sex-specific difference in feed efficiency under HFD conditions. While feed efficiency was comparable between male WT and NT-PGC-1α^−/−^ mice, female NT-PGC-1α^−/−^ mice exhibited decreased feed efficiency compared to WT mice; this was closely associated with increased excretion of dietary fat in feces, although fecal loss of energy needs to be further determined by bomb calorimetry, and reduced uptake of fatty acids by the small intestine and adipose tissue. Accordingly, the decreased energy intake fully accounts for decreased weight gain in male NT-PGC-1α^−/−^ mice, whereas the incomplete digestion and inefficient absorption of dietary fat, along with the decreased fatty acid uptake in adipose tissue, likely contribute to the greater resistance of NT-PGC-1α^−/−^ female mice to HFD-induced obesity. It seems that sex-specific phenotypes are not simply explained by the sex-dependent difference in NT-PGC-1α expression (see Supplementary Fig. S2 online). Further study will be needed to determine if PGC-1α and/or NT-PGC-1α transcriptional activity is differentially regulated by sex hormones. Interestingly, we found that expression of Ob-Rb, a main signaling isoform of the leptin receptor in the hypothalamus^27,28^, is elevated in NT-PGC-1α^−/−^ hypothalamus compared to WT controls (see Supplementary Fig. S3 online). Further investigation will be necessary to evaluate if reduced food intake in NT-PGC-1α^−/−^ mice under HFD feeding is associated with increased leptin signaling in the arcuate nucleus of the hypothalamus.

Although the mechanism for elevated fecal excretion of triglycerides in HFD-fed NT-PGC-1α^−/−^ female mice remains to be determined, decreased intestinal lipid absorption is likely due to reduced uptake of fatty acids by the enterocytes. In line with this, fatty acid translocase CD36 expression was significantly lower in the HFD-fed NT-PGC-1α^−/−^ female jejunum. Interestingly, previous studies have reported sex-, tissue-, and diet-specific regulation of CD36 expression, which is driven by alternative promoter usage in hormone- and nutrition-dependent manners^29–31^. In rat liver, female-dominant expression of CD36 is mediated preferentially through the alternative exon 1a promoter in an estrogen-dependent manner^29,31^. While intestinal CD36 expression is comparable in chow-fed male and female mice, HFD has been shown to greatly increase CD36 expression in females compared with males^32,33^. Intriguingly, PGC-1α has been implicated in fatty acid transport in skeletal muscle^34–36^. Our in vitro data further demonstrated NT-PGC-1α-dependent regulation of CD36 expression in 3T3-L1 adipocytes (Fig. 5C). Moreover, we indeed identified CD36 as one of the PGC-1α and NT-PGC-1α target genes in brown adipose tissue in our previous ChIP-seq assays using a highly specific PGC-1α and NT-PGC-1α antibody^8^ (see Supplementary Fig. S4 online). As a potential mechanism for decreased CD36 gene expression in the HFD-fed NT-PGC-1α^−/−^ female jejunum, we speculate that NT-PGC-1α, which is a relatively stable protein compared to the short-lived PGC-1α protein whose stability is highly dependent on various signaling^1,37^, may be a major transcriptional coactivator under the HFD condition in co-activating sex- and HFD-regulated transcriptional factor(s). In addition to the jejunum in HFD-fed NT-PGC-1α^−/−^ female mice, CD36 gene expression was also decreased in the liver and inguinal WAT, where CD36 expression are shown to be elevated by HFD with greater effects in females^29,31,32^. Further investigation will be needed to identify transcription factor(s) involved in the sex- and HFD-specific regulation of CD36 expression.

Digestion and absorption of dietary lipids, packaging of those lipids into chylomicron particles for secretion into the circulation, and delivery to various tissues for storage or utilization are important steps in maintaining whole body lipid homeostasis^19,38^. The present study identifies NT-PGC-1α as an important regulator of whole body lipid homeostasis under HFD conditions. HFD-fed NT-PGC-1α^−/−^ female mice exhibited lower serum TG levels than WT mice prior to and after an oral fat challenge. NT-PGC-1α deficiency in the jejunum of HFD-fed female mice not only decreased CD36 gene expression but also diminished the expression of genes critical for intestinal triglyceride synthesis and chylomicron production and secretion. Previous studies have reported that CD36 deficiency subsequently impairs induction of genes of intestinal chylomicron formation and secretion^25,26^. Thus, it might be possible that reduced fatty acid uptake by NT-PGC-1α^−/−^ enterocytes subsequently affects synthesis and transport of triglycerides from enterocytes into the blood circulation. This may explain why HFD-fed NT-PGC-1α^−/−^ female mice display lower serum TG levels than WT mice, despite reduced uptake of fatty acids in adipose tissue and liver. However, further investigation will be needed to clearly determine the effect of NT-PGC-1α ablation on intestinal chylomicron formation and secretion.

In summary, NT-PGC-1α deficiency attenuated diet-induced obesity in HFD-fed female mice by reducing food intake, increasing fecal fat excretion, and decreasing fatty acid uptake in the intestine, adipose tissue, and liver. Our findings highlight the role of NT-PGC-1α in regulating whole body lipid homeostasis under HFD conditions.

## Methods

### Animals and diets

All mouse care and experimental procedures were approved by the Institutional Animal Care and Use Committee of the Pennington Biomedical Research Center. All animal experiments were performed in accordance with relevant guidelines and regulations and our animal study reporting adheres to the ARRIVE guidelines^39^. The total number of animals used in this study was determined using the G*Power (v3.1.9.2)^40^ with a power set at 80% and a significance level set at 0.05 for detecting a difference between two groups. NT-PGC-1α^−/−^ mice have been described previously^12^. All mice were housed on a 12-h light/12-h dark cycle. For the cohort 1, 5-week-old male and female NT-PGC-1α^+/+^ (WT littermates) and NT-PGC-1α^−/−^ mice were randomly assigned to two diet groups per genotype and per sex, singly housed, and fed a standard chow diet (13 kcal % fat) (LabDiet, St. Louis, MO, USA) (n = 11 per group) or a HFD (60 kcal % fat) (D12492, Research Diets, Inc., New Brunswick, NJ, USA) (n = 12 per group) at near thermoneutrality (28 °C) for 16 weeks. Their body weight and food intake were monitored every week. Glucose/insulin tolerance tests and indirect calorimetry were carried out at 9–10 and 11–12 weeks of chow or HFD feeding, respectively. If possible, blinding was considered and performed at each step of animal experiments and data analyses to minimize subjective bias. For the cohort 2, 5-week-old female WT and NT-PGC-1α^−/−^ mice (n = 7 per group) were fed a HFD (60 kcal % fat) for 8 weeks and subjected to an oral fat tolerance test. For the cohort 3, 5-week-old female WT and NT-PGC-1α^−/−^ mice (n = 5 per group) were fed a HFD (60 kcal % fat) for 8 weeks and adipose tissue and intestine were collected for fatty acid uptake assays. All mice from cohorts 1–3 were euthanized to collect blood and tissue samples by carbon dioxide asphyxiation followed by cervical dislocation that is in accordance with the established recommendations of the American Veterinary Medical Association (AVMA) Guidelines for the Euthanasia of Animals.

### Metabolic studies

For insulin tolerance test, WT and NT-PGC-1α^−/−^ mice fed a chow or HFD were fasted for 5 h and injected intraperitoneally with insulin (0.75 units/kg BW). For glucose tolerance test, mice were fasted for 16 h and injected intraperitoneally with a glucose bolus (2 g/kg BW for chow-fed group and 1 g/kg BW for HFD-fed group). Blood was collected from the tail vein and blood glucose levels were measured using a Contour Next EZ glucometer (Bayer, Leverkusen, Germany). For oral fat tolerance test, mice were fasted for 16 h prior to receiving a 0.3 ml oral gavage of olive oil. Blood was collected by tail bleeding using a Microvette^®^ 100 K3E (Sarstedt, Numbrecht, Germany) for measurements of serum triglyceride content at the indicated time points. Serum insulin, leptin and triglyceride levels were measured with a Rat/Mouse Insulin ELISA kit (Millipore, Billerica, MA, USA), a Rat/Mouse leptin ELISA kit (Cayman Chemical, Ann Arbor, MI, USA), and Triglyceride Colorimetric Assay kit (Cayman Chemical, Ann Arbor, MI, USA), respectively.

### Body composition and indirect calorimetric analysis

Body composition was determined by TD-NMR using a Bruker Minispec Mouse Analyzer (Bruker Optics, Billerica, MA, USA). To conduct metabolic phenotyping, WT and NT-PGC-1α^−/−^ mice fed a chow or HFD for 11 weeks were acclimated in indirect calorimetry chambers for a week and their body composition was determined prior to transfer into indirect calorimetry chambers. Each mouse was monitored for VO_2_ and VCO_2_ using TSE Systems (TSE systems, Inc., Chesterfield, MO, USA). Energy expenditure (EE) was calculated as (VO_2_ × [3.815 + (1.232 × RER)] × 4.187) and expressed as kilojoules per hour. Locomotor activity and food intake were simultaneously measured while the mice were in the chambers.

### Histological analysis

Tissue samples were fixed in 10% neutral-buffered formalin, embedded in paraffin, and sectioned (5 µm) by the Cell Biology & Bioimaging Core at Pennington Biomedical Research Center. Hematoxylin and eosin-stained paraffin sections of tissue samples were scanned using a Hamamatsu NanoZoomer slide scanner (Hamamatsu, Japan) and viewed through the NDP.view 2 software (https://www.hamamatsu.com/us/en/product/type/U12388-01/index.html).

### Isolation of primary enterocytes

Primary enterocytes were isolated as described previously^41^. Briefly, small intestine was excised and flushed with PBS. The jejunum fragment was dissected, everted, cut into 3–4 cm pieces, and washed five times in Hank’s Balanced Salt (HBS) solution containing 1% FBS and once in Ca^2+^- and Mg^2+^-free HBS solution supplemented with 2% glucose and 2% fatty acid-free BSA. Jejunum pieces were then incubated in isolation buffer (Ca^2+^- and Mg^2+^-free HBS solution supplemented with 0.5 mM DTT and 1.5 mM EDTA) at 37 °C for 15 min with agitation. The resultant cell suspension was passed over a mesh filter and jejunum pieces were reincubated in isolation buffer. Cells collected from the first and second isolation were pooled.

### Fatty acid uptake assay

Uptake of [^14^C]-palmitic acids by tissue explants and cells was measured as described previously^42^ with slight modification. In short, tissue pieces or cells were incubated in HBS solution containing 0.1% fatty acid-free BSA and [^14^C]-palmitic acids (2 µM) at 37 °C for 15 min or on ice followed by washing four times with ice-cold HBS solution containing 0.1% fatty acid-free BSA. The tissue pieces and cells were then lysed in RIPA buffer and the supernatant was used for liquid scintillation counting. The small amount of extracellular [^14^C]-palmitate remaining was corrected from the [^14^C]-palmitate content in samples. Cellular [^14^C]-palmitate uptake was normalized to total protein content in each sample.

### Triglyceride analysis

Tissue samples were homogenized in the Standard Diluent Assay Reagent provided by a Triglyceride Colorimetric Assay kit (Cayman Chemical, Ann Arbor, MI, USA) and triglyceride levels were quantified based on reference standards as described in the manufacturer’s instructions. Triglycerides in fecal lipid extract were determined using the same kit and normalized to fecal weight.

### Cell culture

Murine 3T3-L1 preadipocytes were transduced with retroviruses expressing pBABE empty vector or NT-PGC-1α^5^ and induced for differentiation as described previously^43^.

### Quantitative real-time PCR analysis

Total RNA from tissues was reverse-transcribed for quantitative real-time PCR analysis as described previously^2,4^. Relative mRNA expression of the genes of interest was determined using primers (Table 1) after normalization to cyclophilin by the 2^−ΔΔCt^ method. Primer sequences were obtained from the PrimerBank public resource^44^.

**Table 1:** A list of primer sequences used in this study.

Genes	Forward (5′ to 3′)	Reverse (5′ to 3′)
ACC1	CAGGATCAGCTGGGATACTGAGT	CTCACCCAACCCAGAAAGGCCAA
ACC2	ACCCAACTCTGAAGGGGACC	TCCGAGTCTCCACAGCAATC
ACSL1	TGCCAGAGCTGATTGACATTC	GGCATACCAGAAGGTGGTGAG
ACSL5	AACCAGTCTGTGGGGATTGAG	CGTCTTGGCGTCTGAGAAGTA
aP2	GAAGTGGGAGTGGGCTTTG	ATCCCACTTCTGCACCTGC
ApoA4	CCCGGGCTGAGGTCACTT	GCATTGTTGCTTAGCTGGGTAA
ApoB48	TGAATGCACGGGCAATGA	GGCATTACTTGTTCCATGGTTCT
ATGL	AGCATCTGCCAGTATCTGGTGAT	CACCTGCTCAGACAGTCTGGAA
CD11b	CCACACTAGCATCAAGGGCA	CCCTGATCACCGTGGAGAAG
CD11c	CTGTCATCAGCAGCCACGA	ACGGGACTCTTCTGCATGTG
CD274	GCTCCAAAGGACTTGTACGTG	TGATCTGAAGGGCAGCATTTC
CD36	GCCAAGCTATTGCGACATGA	TCTCAATGTCCGAGACTTTTCA
CPT1β	TCTAGGCAATGCCGTTCAC	GAGCACATGGGCACCATAC
CPT2	CCGAGGCATTTGTCAGGGAG	AAGTGTCGGTCAAAGCCCTG
Cyclophilin	CCATCGTGTCATCAAGGACTTCAT	CTTGCCATCCAGCCAGGAGGTCTT
DGAT1	CGTGGGCGACGGCTACT	TGAGCTGAACAAAGAATCTTGCA
DGAT2	ATCTTCTCTGTCACCTGGCT	ACCTTTCTTGGGCGTGTTCC
F4/80	TGGGATGTACAGATGGGGGA	TCCTGGGCCTTGAAAGTTGG
FABPpm	GGACCTCCAGATCCCATCCT	GGTTTTCCGTTATCATCCCGGTA
FASN	TCCTGGAACGAGAACACGATCT	GAGACGTGTCACTCCTGGACTTG
FATP1	TCTGTTCTGATTCGTGTTCGG	CAGCATATACCACTACTGGCG
FATP2	GATGCCGTGTCCGTCTTTTAC	GACTTCAGACCTCCACGACTC
FATP4	GATGGCCTCAGCTATCTGTGA	GGTGCCCGATGTGTAGATGTA
HSL	TGAGATGCCACTCACCTCTG	GCCTAGTGCCTTCTGGTCTG
I-FABP	GTGGAAAGTAGACCGGAACGA	CCATCCTGTGTGATTGTCAGTT
IL-1β	CAACCAACAAGTGATATTCTCCATG	GATCCACACTCTCCAGCTGCA
IL-6	ATGGATGCTACCAAACTGGAT	TGAAGGACTCTGGCTTTGTCT
LCAD	GCCCGATGTTCTCATTCTGGA	TGCTTGCCAGCTTTTTCCCAG
L-FABP	GGGGGTGTCAGAAATCGTG	CAGCTTGACGACTGCCTTG
LPL	CTTTCACTCGGATCCTCTCG	AGGTGGACATCGGAGAACTG
MCAD	CAATGATGTGTGCTTACTG	CCAGTAAAGGCTTTACTAGC
MGAT2	TGGGAGCGCAGGTTACAGA	CAGGTGGCATACAGGACAGA
MGL	TGATTTCACCTCTGGTCCTTG	GTCAACCTCCGACTTGTTCC
MTTP	CTCTTGGCAGTGCTTTTTCTCT	GAGCTTGTATAGCCGCTCATT
NOS2	GTTCTCAGCCCAACAATACAAGA	GTGGACGGGTCGATGTCAC
NPY	ATGCTAGGTAACAAGCGAATGG	TGTCGCAGAGCGGAGTAGTAT
NT-PGC-1α	TGCCATTGTTAAGACCGAG	GGTCACTGGAAGATATGG
Ob-Rb	ACTCTGGTCAGCAACGATAAACTA	GAAAAATGTCTGGGCCTCTGTCTC
Perilipin	GGCCTGGACGACAAAACC	CAGGATGGGCTCCATGAC
PGC-1α	TGCCATTGTTAAGACCGAG	TTGGGGTCATTTGGTGAC
PPARγ	AGGCCGAGAAGGAGAAGCTGTTG	TGGCCACCTCTTTGCTCTGCTC
POMC	ATGCCGAGATTCTGCTACAGT	TCCAGCGAGAGGTCGAGTTT
SCAD	ACCAAAGCTTGGATCACCAACTCC	AACCAGGAAGGCACTGATACCCTT
SCD1	GTGCCGTGGGCGAGGGCTTC	AGCCCAAAGCTCAGCTACTCTT
TNF4α	CCCTCACACTCAGATCATCTTCT	GCTACGACGTGGGCTACAG

### Statistical analysis

All line and bar graphs were created by using the Prism 6 software (GraphPad Software, San Diego, CA, USA), and student *t* test or two-way ANOVA was used to compare the differences between groups using the Prism 6 software. Data are presented as mean ± SEM. Values of *P* < 0.05 were considered statistically significant.

## Supplementary Information

Supplementary Information

## Data Availability

All data generated during this study are included in this published article and its Supplementary Information file.
