# Supplementary material for: NT-PGC-1α deficiency attenuates high-fat diet-induced obesity by modulating food intake, fecal fat excretion and intestinal fat absorption
Source: Sci Rep. 2021 Jan 14;11:1323. doi: 10.1038/s41598-020-79823-9 (PMC7809341; doi:10.1038/s41598-020-79823-9)
Supplement: Supplementary file 1 — Supplementary Information [file 41598_2020_79823_MOESM1_ESM.pdf]

NT-PGC-1α deficiency attenuates high-fat diet-induced obesity by modulating food intake, fecal fat excretion and intestinal fat absorption

Jihyun Kim[1]*, Jiyoung Moon[1]*, Chul-Hong Park[1], Jisu Lee[1], Helia Cheng[1], Z. Elizabeth Floyd[2],

Ji Suk Chang[1]

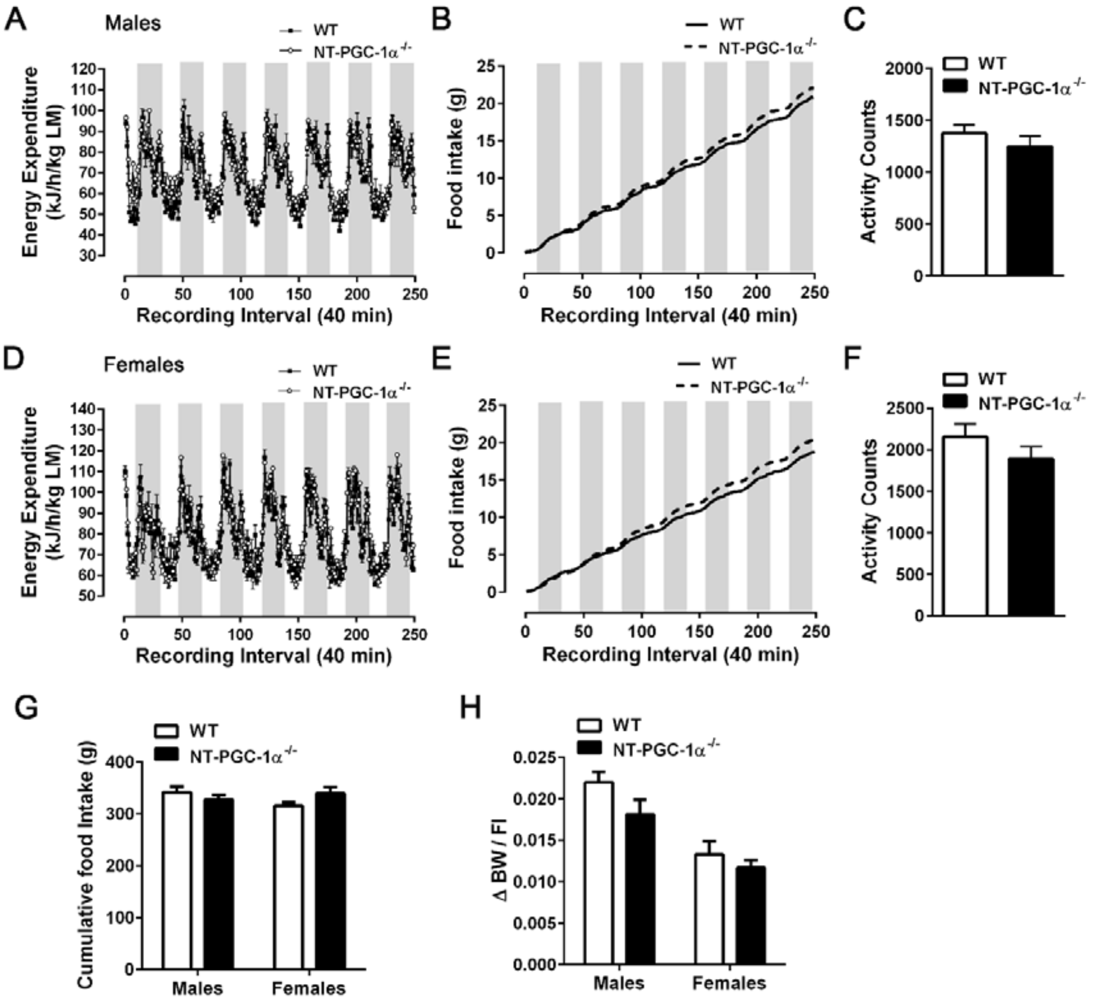

Supplementary Figure S1. Caloric intake and energy metabolism in NT-PGC-1α$^{-/-}$ mice fed a chow diet. (A-F) Energy expenditure, food intake, and average locomotor activity of male and female WT and NT-PGC-1α$^{-/-}$ mice fed a chow diet (n=11 per group). (G) Cumulative food intake of male and female WT and NT-PGC-1α$^{-/-}$ mice during chow feeding (n=11 per group). (H) Cumulative weight (g) gained over 16 weeks divided by the cumulative food intake (g/mouse) over the same period on chow (n=11 per group).

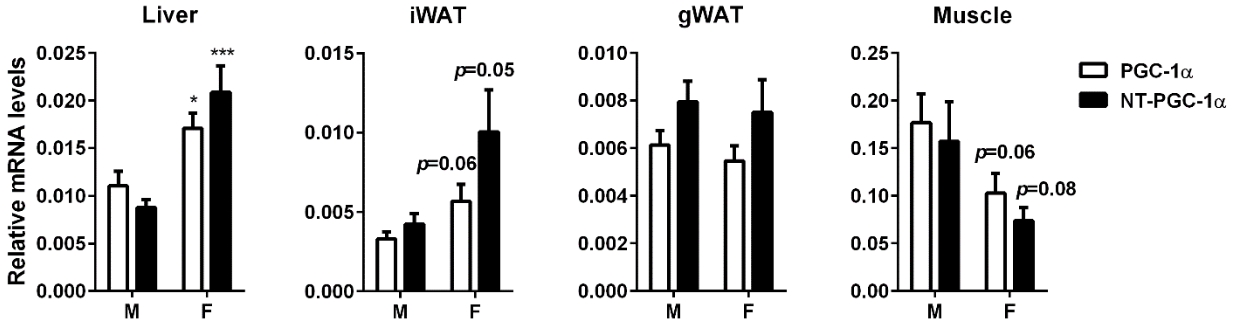

Supplementary Figure S2. Differential expression of PGC-1α and NT-PGC-1α in a sex- and tissue-specific manner. Quantitative qPCR analysis of PGC-1α and NT-PGC-1α in liver, iWAT, gWAT, and muscle from HFD-fed male and female WT mice (n=10-12 per group). Data are presented as the mean ± SEM. *$P < 0.05$, ***$P < 0.001$ determined by Student's *t* test.

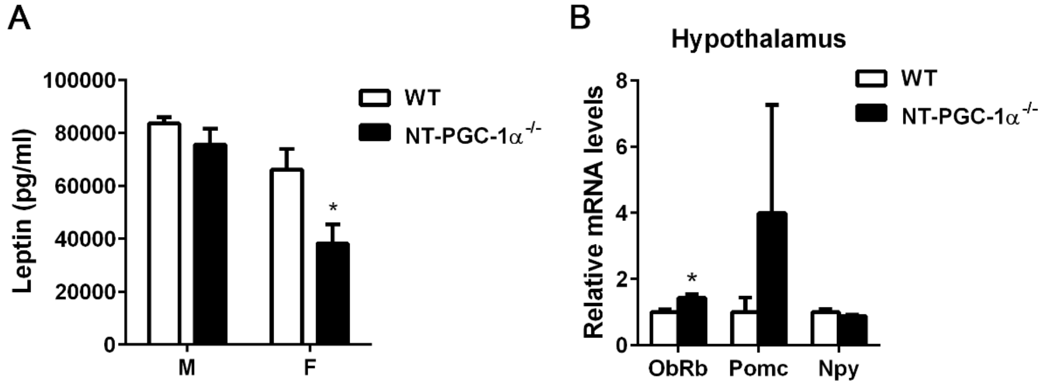

Supplementary Figure S3. Effect of NT-PGC-1α ablation on circulating leptin levels and hypothalamic gene expression. (A) Serum leptin levels in HFD-fed male and female WT and NT-PGC-1α$^{-/-}$ mice (n=10 per group). (B) Quantitative qPCR analysis of hypothalamic genes in HFD-fed female WT and NT-PGC-1α$^{-/-}$ mice (n=7 per group). All data are presented as the mean ± SEM. *P < 0.05 determined by Student's *t* test.

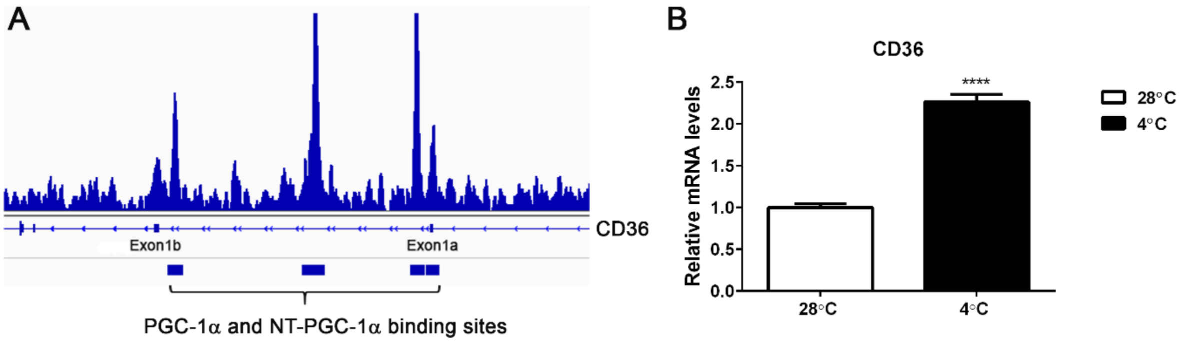

Supplementary Figure S4. Chromatin occupancy of PGC-1α and NT-PGC-1α at the CD36 gene promoters in brown adipose tissue. (A) ChIP-seq with PGC-1α antibody recognizing both PGC-1α and NT-PGC-1α. Four small boxes represent the binding sites for PGC-1α and/or NT-PGC-1α at the CD36 gene promoters in cold-activated BAT. (B) Enrichment of PGC-1α and NT-PGC-1α at the CD36 gene promoters is accordant with a cold-dependent increase in CD36 gene expression in BAT (n=6 per group). Data are presented as the mean ± SEM. ****$P <$ 0.0001 determined by Student's *t* test.
